# Validation of Psychometric Properties of the Nursing Work Index—Revised Scale in Portugal

**DOI:** 10.3390/ijerph19094933

**Published:** 2022-04-19

**Authors:** Sara Anunciada, Patrícia Benito, Filomena Gaspar, Pedro Lucas

**Affiliations:** 1 Nursing Research, Innovation and Development Centre of Lisbon (CIDNUR), Nursing School of Lisbon, Av. Prof. Egas Moniz, 1600-096 Lisbon, Portugal; mfgaspar@esel.pt (F.G.); prlucas@esel.pt (P.L.); 2 Central Lisbon Health Centres, Rua Carvalho Araújo, nº 103-7º, 1900-181 Lisbon, Portugal; 3 Barreiro Montijo Hospital Centre, 2830-003 Barreiro, Portugal; pbenito88@gmail.com

**Keywords:** evidence-based practice, health facility environment, hospitals, instrument development, nursing, quality of care, practice management, psychometrics, validation studies, work environment

## Abstract

The use of instruments designed to assess the nursing practice environment is crucial to improve the quality of nursing care, to anticipate problems and difficulties that may arise in organizations, and allow nurse managers to implement changes and improvements in key areas. In this study, we aimed to evaluate the psychometric properties of the Nursing Work Index—Revised Portuguese version (NWI-R-PT) scale. A quantitative, observational, descriptive, and cross-sectional study was conducted. Methods: The sample consisted of 767 nurses from 4 public Portuguese hospitals. Exploratory and confirmatory factor analysis techniques were used to test the distinct structural models. The scale’s accuracy was evaluated through internal consistency, using Cronbach’s alpha. Results: NWI-R-PT internal consistency was 0.91. The NWI-R-PT model with six factors, namely “Management Support,” “Professional Development,” “Fundamentals of Nursing,” “Nurse–Physician Relationship,” “Endowments,” and “Organization of Nursing Care,” was supported by exploratory and confirmatory factor analysis. The NWI-R-PT scale presents adequate goodness-of-fit indices concerning the final factorial model and the convergent validity. Conclusions: The NWI-R-PT scale has a competent and reliable structure. The scale’s validity is confirmed; therefore, it may be employed in all contexts in clinical practice, research, and nursing management. The NWI-R-PT is a useful and valid instrument to assess the nursing environment in hospitals, primary care, long-term care, and nursing homes. The scale has significance in improving the quality of nursing care and patient safety, the professional development of nurses, and organizational results.

## 1. Introduction

The nursing practice environment is critical for health system success [1,2,3]. It is related to the quality of care, job satisfaction, patient safety, and effective care of clients and is essential for an organization’s efficiency [2,3,4,5,6,7]. According to Lake [6], the nursing practice environment can be described as the organizational attributes of a working ambience that promote or constrict a nurse’s practice.

The promotion of the quality of the services provided by the nursing team and their contribution to improving the contexts of professional practices are key elements of the nursing practice environment [2,3]. The quality of care is a crucial element in the profession and refers, among other aspects, to the direct relationship between the patient and the nurse, which is dependent on many factors, especially the nursing practice environment [2,3,7,8].

A favorable nursing practice environment leads to enhanced patient outcomes, which is an important factor to develop nurses’ satisfaction [6,9] as well as to retain nursing staff [2,3,5,7]. A favorable nursing practice environment is defined by the adequacy of manpower and resources, the effective participation of nurses in the governance of organizations, the quality of care, the provision of nursing care, and good relations between the different professional teams of nursing services [1,2,3,6]. 

A safe nursing practice environment is characterized by good working relationships, governance support of professionals, and a good work–life balance [2,3,7,10,11]. 

Nursing practice environment has an impact on patient and nurse outcomes and environments where nurses are autonomous [12]. Environmental control and positive interactions between the nurses and the medical team reduce burnout levels [13,14,15,16,17,18,19,20], increase job satisfaction [1,5,13,14,21,22,23,24], lower intention to quit [5,7,9,10,13,17,18,21], and ensure better results for clients with regard to the quality of care [1,7,10,13,15,21,25,26,27] and patient safety [1,7,13,16,17,19,21,25].

In this sense, it is important to adjust the composition, principles, and methods of institutions because of their impact on the nursing practice environment. Monitoring the nursing practice environment to minimize its imperfections will permit the execution of continuous improvement processes, in turn improving health-related outcomes. In these circumstances, the employment of instruments developed to assess the nursing practice environment is crucial to improve the quality of care [1,7,28,29,30,31] and to anticipate problems and difficulties that may arise in organizations.

To understand how composition, principles, and methods of institutions affect nursing outcomes, Aiken and Patrician [12], based on the NWI and a literature review, developed the NWI-R into a 57-item scale of models of professional practice, theoretically based on the sociology of organizations, professions, and work [6,12].

In recent decades, several studies have identified different factor structures in the NWI-R in the United States [6,32,33,34] and Canada [35], and translated versions of the instrument have been tested and validated in Canada [36], Brazil [37], Iceland [38], the United Kingdom [39], Belgium [40], Lebanon [41], and South Korea [42,43]. Most studies found solid factor structures with some similarities to the original scale.

## 2. Materials and Methods

### 2.1. Aim

The aim of the present study was to analyze the psychometric properties of the NWI-R-PT scale using factor analysis. The sample consisted of nurses from several hospitals in Portugal.

### 2.2. Study Design

This study is quantitative, observational, descriptive, and cross-sectional. 

### 2.3. Participants

This study sample consisted of 767 nurses who worked in public hospitals.

### 2.4. Translation and Cultural Adaptation

After authorization of the original scale [12] by the authors and using the methodology proposed by reference authors [44,45], two uncommitted translators converted the scale to Portuguese. One of the translators was bilingual, fluent in Portuguese but with English as their mother tongue, with knowledge in the field of nursing, and the other was a professional translator without knowledge in the field of health. The translation and adaptation consisted of an exhaustive analysis not only based on the lexical dimension, but also on the cultural adaptation to the Portuguese reality, to ensure that the items kept the meaning identical to that of the original language. Then, the two versions were analyzed, resulting in a consensus version. 

From the consensus version, in a third stage, the back-translation was performed by a third professional translator who was unaware of the original version, giving rise to the back-translated version. 

The translation and back-translation steps were conducted without difficulty. At the meeting with the group of experts, some modifications were made to guarantee the equivalences—semantic, idiomatic, cultural, and conceptual—of the translated instrument with the original instrument.

Then, given the scale obtained, the comprehension test or pre-test was performed, which was applied to a non-probabilistic sample of 35 nurses. The sample size was selected based on the results of Perneger et al. [46], which focused on the mathematical analysis of the necessary sample size to carry out the pre-test with some confidence, considering the prevalence of problems with items of a given scale. It revealed that a minimum of 22 individuals was needed to detect 90% of the scale’s problems [46]. If more than 20% of the sample was doubtful concerning the scale, it would be necessary to re-analyze and probably translate it [44,45]. 

The scale was answered by 35 nurses, and the average time was 10 min. Participants did not find any problem with the scale when filling it out. They considered it time-consuming to complete, but its content was clearly perceived.

### 2.5. Data Collection

Data were collected between 2017 and 2019 in four public hospitals in Portugal after the NWI-R translation and adaptation process.

Authorization was requested from the ethics committees of the hospitals. Contacts were made with hospital nurses.

From the translation and cultural adaptation, the NWI-R had 54 items, evaluated by a Likert-type scale of 5 response options.

The sociodemographic characterization consists of age, gender, and professional nursing experience and academic background.

### 2.6. Ethical Considerations

Data were collected after approval from the Hospital Ethics Committees. We guaranteed the confidentiality and anonymity of participants and data at all stages of the study. All participants were fully informed about the study, participated voluntarily, and were free to withdraw at any time.

### 2.7. Data Analysis

The variables were subject to a comparative and descriptive analysis. Continuous variables are reported as means and standard deviations. The categorical variables are reported by absolute and relative frequency.

Different structural models were investigated using exploratory factor analysis and confirmatory factor analysis techniques.

To carry out the exploratory factor analysis, the principal component methods were used with Varimax rotation. To analyze the adequacy of the data for the exploratory factor analysis, two tests were utilized: the Kaiser–Meyer–Olkin (KMO) test and Bartlett’s sphericity test, both of which indicate the adjustment of the data to the factor analysis [47].

The Kaiser criterion for factor extraction and the Varimax rotation were used. The entire explained variance of the results has been subject to critical analysis.

To assess the quality of the fit of the model obtained through the exploratory factor analysis, confirmatory factor analysis was performed with the software AMOS, version 26.0, IBM. The confirmatory factor analysis was performed using the methodology of maximum likelihood, which implies autonomy of observations; multivariate normal distribution, analyzed by kurtosis (Ku) coefficients and asymmetry (Sk); and no outliers, evaluated by Mahalanobis square distance D^2^ [48].

To assess the overall adequacy of the model in the confirmatory factor analysis, the following indices were used: goodness of fit indices χ^2^/df, chi-square ratio and degrees of freedom, Comparative Fit Index (CFI), Goodness of Fit Index (GFI), and root mean square error of approximation (RMSEA), with scores between 0.05 and 0.1 considered acceptable [48]. The model fit was considered good for CFI and GFI values above 0.9 and RMSEA values below 0.10 [48,49]. The validity of the scale was calculated through two subcomponents: the convergent validity, calculated by the average variance extracted (AVE) and composite reliability (CR) for each dimension, considering AVE ≥ 0.50 and FC ≥ 0.70; and by the factorial validity, considering standardized factor loadings λ and individual reliability λ2, which are also indicators of the quality of the adjustment, with minimum values of 0.50 and 0.25, respectively [48]. The models with low MECVI Index proved to be more suitable and were used for the modification indices foreseen in the AMOS and in the theoretical considerations [48].

All analyzes were performed with statistical software SPSS version 25.0 and software AMOS version 26, SPSS, IBM Inc., Chicago, IL, USA.

### 2.8. Validity and Reliability/Rigor

The instrument’s internal consistency and reliability were determined using an alpha coefficient (α) and composite reliability. The range is from 0 to 1, with 0.70 being the value for adequate reliability [47].

The scale structural validity was first tested through exploratory factor analysis and then using structural equation modeling, and through confirmatory factor analysis with factorial and convergent validity. After multivariate normality was assumed, the factor validity was tested using maximum likelihood estimation. The proposed factor model was considered valid when factor weights were greater than 0.4 for each of the items [47,50,51]. Construct validity was calculated using convergent and discriminant validity. The former was calculated by AVE for each factor, considering the minimum value as 0.50. Discriminant validity, on the other hand, was established by confirmation that the AVE for each is equal to or greater than the square of the correlation between the two factors.

## 3. Results

As for the characterization of the sample of 767 hospital nurses, 84.2% were female.

The mean age was 36.8 years (9.5 years).

Nurses with an academic degree were 85.2%, and 14.8% had postgraduate training (master’s: 10.7%; specialization: 3.7%; postgraduate: 0.3%; Ph.D.: 0.1%).

Specialist nurses were 13.6% of the sample and 2.8% of nurse managers.

The average time in the profession was 8.8 years (8.9 years).

### 3.1. Exploratory Factor Analysis

Bartlett’s test of sphericity was relevant (*p* < 0.001). The KMO index presented a value of 0.94, a remarkable recommendation value concerning the analysis of principal components [47].

The NWI-R exploratory factor analysis identified 6 components that provide a rationale for 53.2% of the total variance. In exploratory factor analysis, 23 items were eliminated for not having met the factor loading criteria. The final presentation contains 31 items in 6 factors, named according to a conceptual perspective. The 6 identified components are Management Support with 8 items; Professional Development with 6 items; Fundamentals of Nursing with 6 items; Nurse–Physician Relationship with 4 items; Endowments with 3 items; and Organization of Nursing Care with 4 items (Table 1).

### 3.2. Reliability Analysis

A Cronbach’s alpha value of 0.91 was reached regarding the reliability analysis for the entire scale, which is adequate and displays a prominent measure of internal consistency. The original scale by Aiken and Patrician [12] obtained an alpha value of 0.96. 

Alpha values for each subscale were greater than 0.59 and are described in Table 2.

### 3.3. Confirmatory Factor Analysis

The six factors of the NWI-R-PT found in our sample were each subjected to confirmatory factor analysis. Although the NWI-R-PT items had good factor results, the confirmatory factor analysis model originally presented inadequate suitability (χ^2^/df = 3.538; CFI = 0.870; GFI = 0.885; RMSEA = 0.058; MECVI = 2.145). 

Additionally, the Mahalanobis distances revealed the existence of diverse multivariate outliers. Thus, a part of the multivariate outliers was removed, and the modification indices were analyzed. 

As the modifications were not noteworthy, the model modification rates were analyzed. The highest rates of modification occur between the correlations of errors in items 4 and 5, items 22 and 23, and items 23 and 24 for a total of 21. When items included in the same factor have related errors, it is typical to add this trajectory to the model, explaining it from a theoretical point of view, by the items’ almost identical formulation or content.

**Figure 1:**
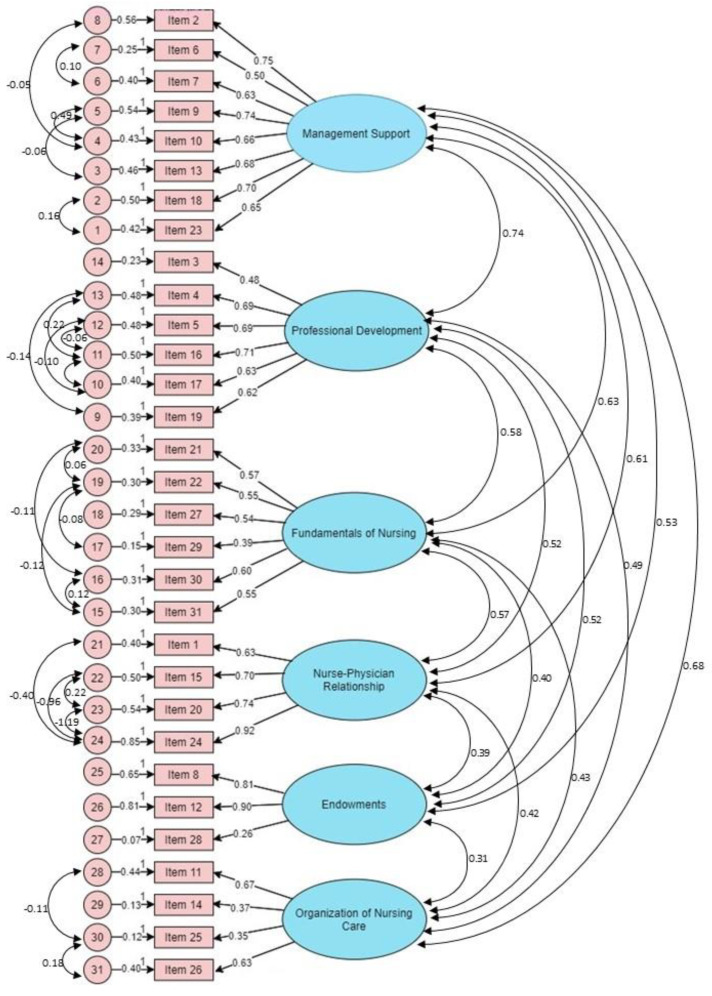
Six-factor model of Nursing Work Index-R in Portugal.

From the review performed, and weighing the differences with the initial model, the model (Figure 1) presented adequate suitability with more fitting indices for the sample and a lower MECVI (1.563 vs. 2.145).

The reliability of the construct presented adequate internal consistency with a prominent measure of internal consistency of the scale and appropriate reproducibility of the total scale (α = 0.91) and of the subscales (Table 2). Assuming AVE ≥ 0.50 [48,52] as an indicator of convergent validity, it was adequate for almost all factors in the scale: Management Support, Professional Development, Fundamentals of Nursing, Nurse–Physician Relationship, Endowments, and Organization of Nursing Care.

The factorial invariance analysis of the model in the two independent subsets, test and validation, revealed adequate goodness of fit indices in the final factorial solution (χ^2^/df = 2.495; CFI = 0.927; GFI = 0.923; RMSEA = 0.044; MECVI = 1.563).

## 4. Discussion

The results provided empirical evidence indicating a satisfactory psychometric performance of the NWI-R in the sample of Portuguese nurses. The model, with six components, is conceptually consistent and in general agreement with the suggestions of the original scale’s author [12] and obtained support from both an exploratory and a confirmatory perspective. Nevertheless, the items in each of the components are different when compared with studies from abroad, which can be justified by the differences in culture, health system models, organizational models, or the profession, which can affect nurses’ responses.

Although some components have low alpha values, the global Cronbach’s alpha of the scale is high, similarly to what is observed in other studies [32,37,38,41,42,43], which also obtained low Cronbach’s alpha values in their components, but high values in the alpha of the scale. The NWI-R-PT scale evaluated in this study has 31 items instead of 57 in the original scale. 

Other studies have adjusted the NWI-R to other factor structures, depending on cultural contexts, with different compositions [32,33,34,38,39,40,41,42,43]. Consistent with the factor analyses performed by Cho et al. [42], Choi et al. [32], Erickson et al. [33], Estabrooks et al. [35], Gunnarsdóttir et al. [38], Kim et al. [43], Lake [6], Li et al. [34], McCusker et al. [36], Slater and McCormack [39], and Van Bogaert et al. [40], the factor analysis of the present study did not identify the autonomy, control over practice, and organizational support dimensions used in several previous studies [12,37,41]. 

The confirmatory factor analysis revealed that the six-factor model is the most solid and is suitable for this large sample of Portuguese data.

All coefficients were significant. The coefficients of the model’s adequacy indices were sufficient and support the structure of the six factors. The invariance analysis of the six-factor model reaffirmed its stability. 

Other studies used a six-factor model similar to ours, characterized by Management Support, Professional Development, Nursing Fundamentals, Nurse–Doctor Relationship, Staffing, and Organization of Nursing Care [41,43]. As for the number of items, other studies also presented comparable scales to the one in the present study [6,38,40,41].

Low factor weights influenced composite reliability and factor validity in some items. Furthermore, the low AVE values found in some dimensions can be justified by the high variability in the factor loadings of the items. 

### Limitations

The target of this study was nurses working in hospital wards. Future research should confirm the applicability of the scale in several settings.

The Cronbach’s alpha values of the dimensions Endowments, Fundamentals of Nursing, and Organization of Nursing Care are borderline. However, in our assessment of the composition of the dimensions, we considered that they correspond to the constructs they assess.

## 5. Conclusions

In this study, we used a sample consisting of Portuguese hospital nurses and revealed an adequate structure of six factors of the NWI-R-PT. This structure showed evidence of reliability and validity and is in general agreement with what was recommended by the authors of the original scale. The totality of the factors had adequate factor loading, and they have a semantic relationship.

The study demonstrates that in the Portuguese context, the Nursing Work Index-R-PT is an important tool to support the decision making of nurse managers, to characterize the nursing practice environment, and to enhance working conditions in organizations. It contributes to the improvement of the nursing practice environment in the most varied contexts where nursing care is provided.

The results revealed the validity of the scale and that it can be a support tool in nursing practice, nursing research, and nursing management. This study demonstrates the global relevance of the use of the Nursing Work Index-R-PT and, moreover, it identifies multiple organizational characteristics in clinical contexts, improving the identification of which work ambiences are favorable or unfavorable to patient safety and the quality of care.

Finally, this study also provides important inputs to the health policies of countries and strategic planning.

## Figures and Tables

**Table 1:** Nursing Work Index-R-PT components.

Items		Components				
	Management Support	Professional Development	Fundamentals of Nursing	Nurse–Physician Relationship	Endowments	Organization of Nursing Care
2	0.75	-	-	-		-
6	0.50	-	-	-		-
7	0.63	-	-	-		-
9	0.74	-	-	-		-
10	0.66	-	-	-		-
13	0.68	-	-	-		-
18	0.70	-	-	-		-
23	0.65	-	-	-		-
3	-	0.45	-	-		-
4	-	0.70	-	-		-
5	-	0.70	-	-		-
16	-	0.71	-	-		-
17	-	0.63	-	-		-
19	-	0.62	-	-		-
21	-	-	0.57			
22	-	-	0.55	-		-
27	-	-	0.54	-		-
29	-	-	0.39	-		-
30	-	-	0.60	-		-
31	-	-	0.55	-		-
1	-	-	-	0.63		-
15	-	-	-	0.70		-
20	-	-	-	0.74		-
24	-	-	-	0.92		-
8	-	-	-	-	0.81	-
12	-	-	-	-	0.90	-
28	-	-	-	-	0.26	-
11	-	-	-	-	-	0.67
14	-	-	-	-	-	0.37
25	-	-	-	-	-	0.35
26	-	-	-	-	-	0.63

**Table 2:** Construct validity of the Nursing Work Index-R-PT.

Components	Alpha	AVE
1. Management Support	0.86	0.45
2. Professional Development	0.81	0.41
3. Fundamentals of Nursing	0.69	0.24
4. Nurse–Physician Relationship	0.77	0.58
5. Endowments	0.65	0.51
6. Organization of Nursing Care	0.59	0.27

## Data Availability

Restrictions apply to the availability of these data. Data were obtained from a third party and are available with the permission of the third party.
